# Supplementary material for: Transcriptomic Insights into Mechanisms of Early Seed Maturation in the Garden Pea (Pisum sativum L.)
Source: Cells. 2020 Mar 23;9(3):779. doi: 10.3390/cells9030779 (PMC7140803; doi:10.3390/cells9030779)
Supplement: Supplementary file 1 [file cells-09-00779-s001.zip › Supplementary materials/FileS4.zip!/FileS3/TFs - Results/Summary Sheets - Groups/Expression Portraits Groups.pdf]

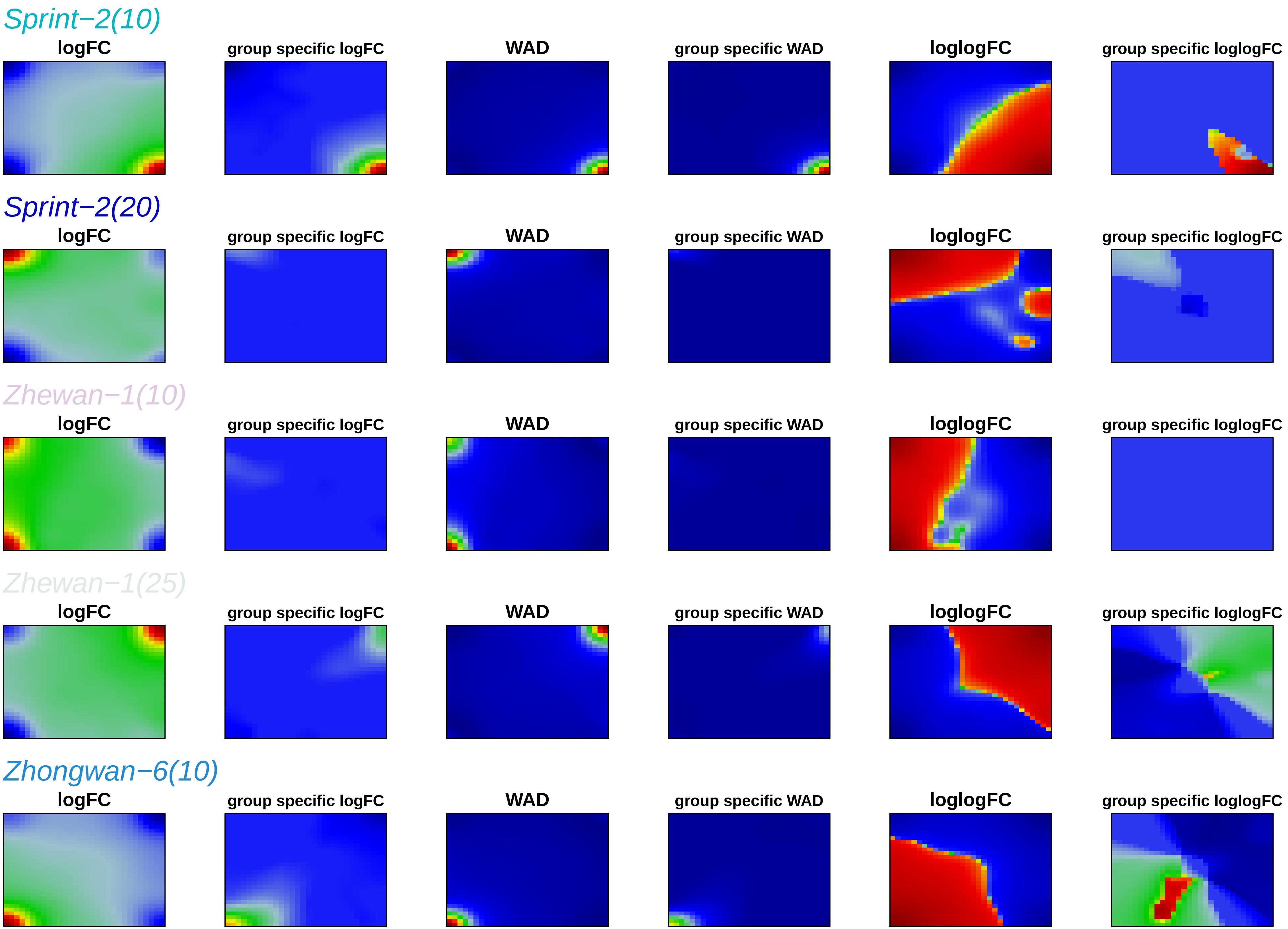
Sprint−2(10)
logFC
group specific logFC
WAD
group specific WAD
loglogFC
group specific loglogFC
Sprint−2(20)
logFC
group specific logFC
WAD
group specific WAD
loglogFC
group specific loglogFC
Zhewan−1(10)
logFC
group specific logFC
WAD
group specific WAD
loglogFC
group specific loglogFC
Zhewan−1(25)
logFC
group specific logFC
WAD
group specific WAD
loglogFC
group specific loglogFC
Zhongwan−6(10)
logFC
group specific logFC
WAD
group specific WAD
loglogFC
group specific loglogFC

Zhongwan−6(25)

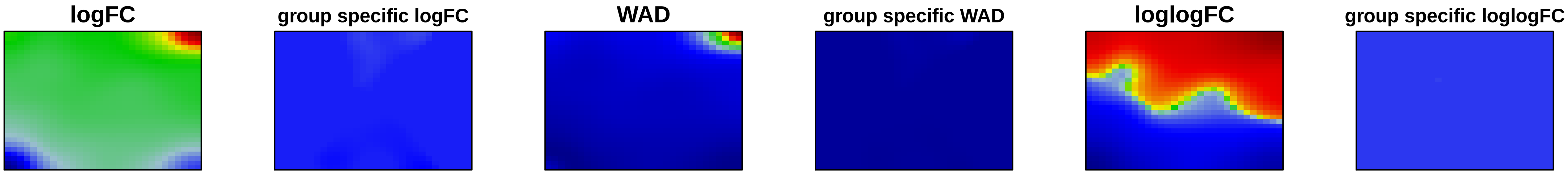

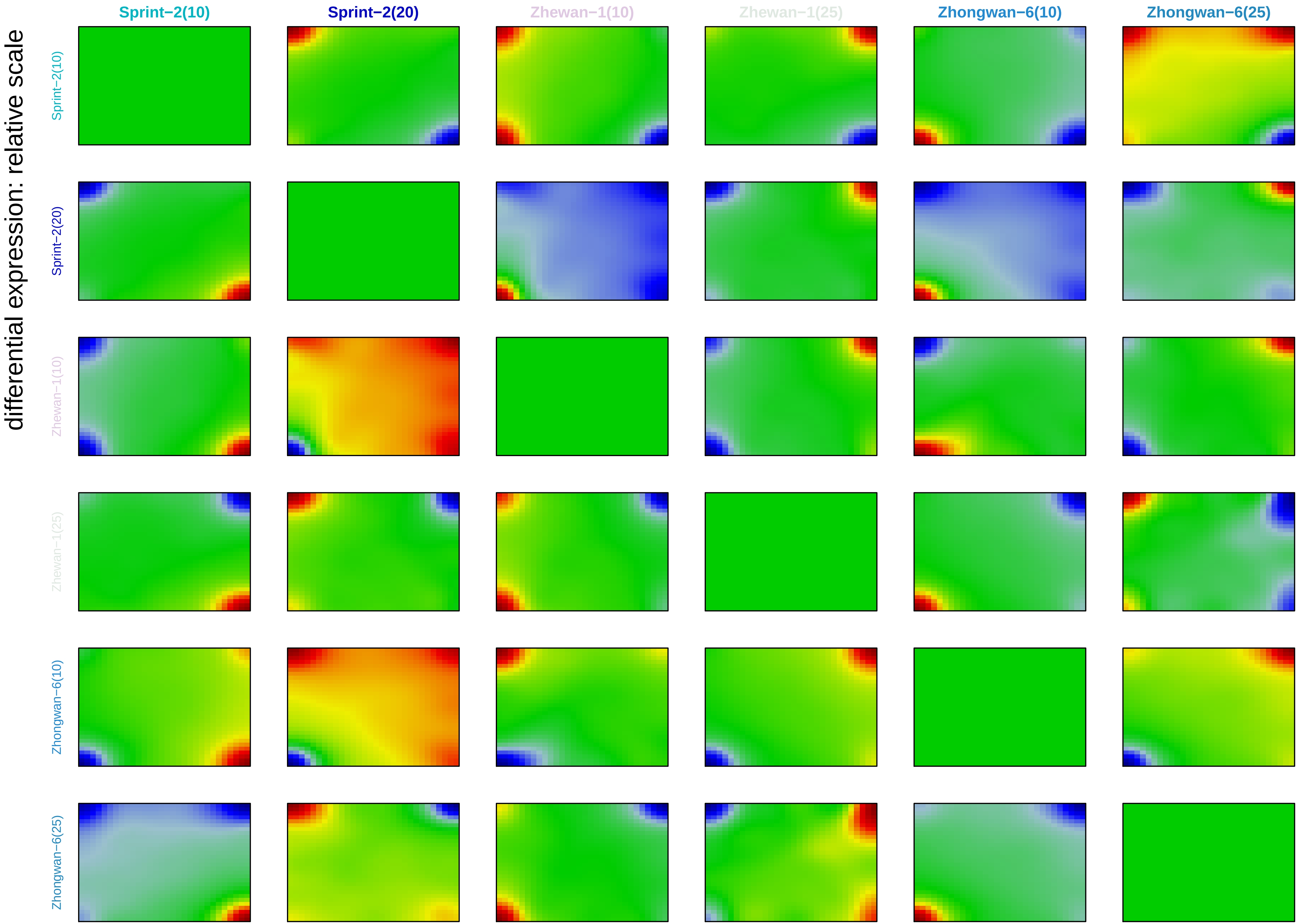
differential expression: relative scale
Sprint−2(10)
Sprint−2(20)
Zhewan−1(10)
Zhewan−1(25)
Zhongwan−6(10)
Zhongwan−6(25)

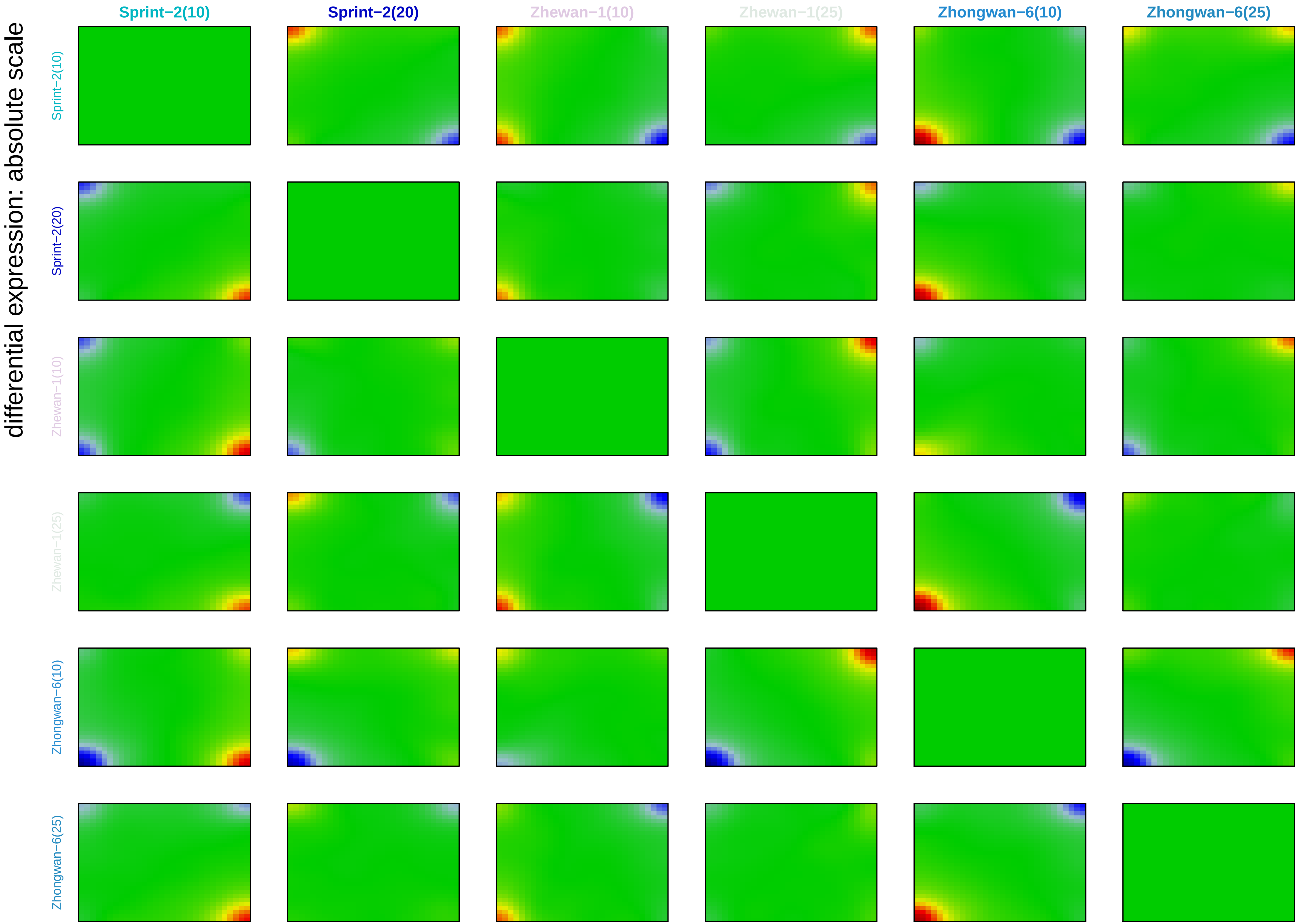
differential expression: absolute scale
Sprint−2(10)
Sprint−2(20)
Zhewan−1(10)
Zhewan−1(25)
Zhongwan−6(10)
Zhongwan−6(25)

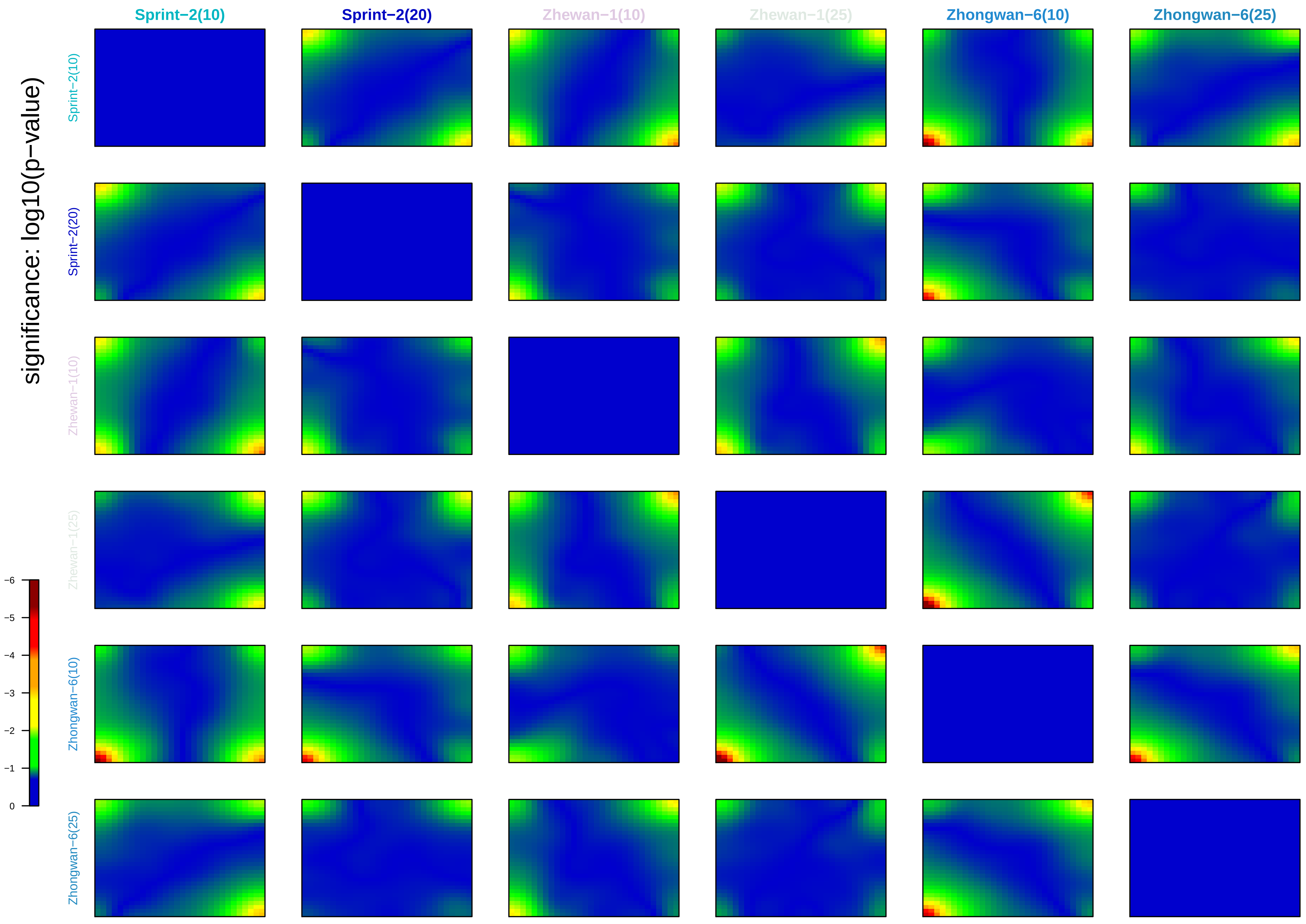

Sprint−2(10)
Sprint−2(20)
Zhewan−1(10)
Zhewan−1(25)
Zhongwan−6(10)
Zhongwan−6(25)
significance: log10(p−value)
Sprint−2(10)
Sprint−2(20)
Zhewan−1(10)
Zhewan−1(25)
Zhongwan−6(10)
Zhongwan−6(25)
−6
−5
−4
−3
−2
−1
0

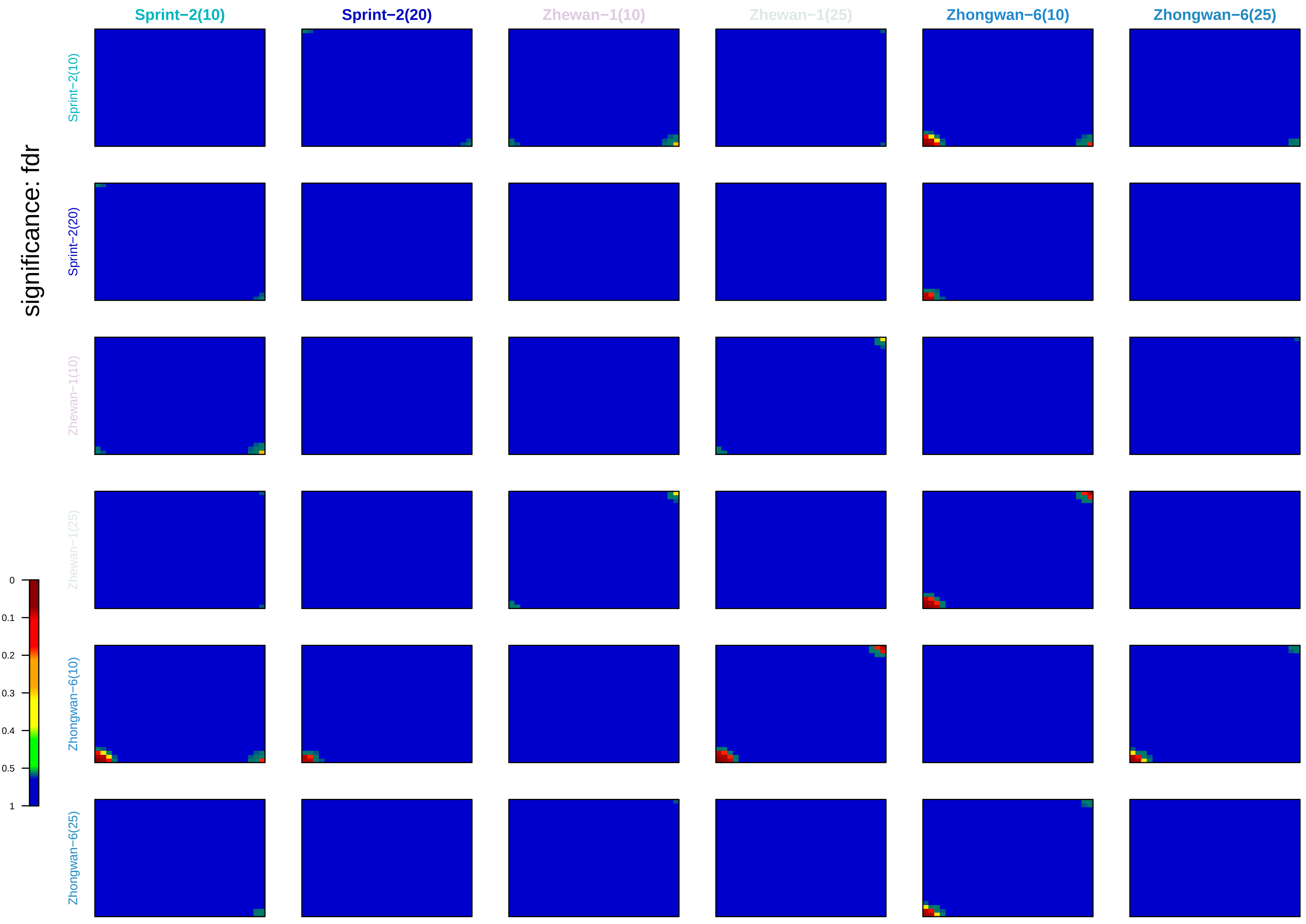

significance: fdr
Sprint−2(10)
Sprint−2(20)
Zhewan−1(10)
Zhewan−1(25)
Zhongwan−6(10)
Zhongwan−6(25)
0
0.1
0.2
0.3
0.4
0.5
1
